# Development of a Halotolerant Community in the St. Lucia Estuary (South Africa) during a Hypersaline Phase

**DOI:** 10.1371/journal.pone.0029927

**Published:** 2012-01-06

**Authors:** Nicola K. Carrasco, Renzo Perissinotto

**Affiliations:** School of Biological and Conservation Sciences, University of KwaZulu-Natal, Westville Campus, Durban, South Africa; Argonne National Laboratory, United States of America

## Abstract

**Background:**

The St. Lucia Estuary, Africa's largest estuarine lake, is currently experiencing unprecedented freshwater deprivation which has resulted in a northward gradient of drought effects, with hypersaline conditions in its northern lakes.

**Methodology/Principal Findings:**

This study documents the changes that occurred in the biotic communities at False Bay from May 2010 to June 2011, in order to better understand ecosystem functioning in hypersaline habitats. Few zooplankton taxa were able to withstand the harsh environmental conditions during 2010. These were the flatworm *Macrostomum* sp., the harpacticoid copepod *Cletocamptus confluens*, the cyclopoid copepod *Apocyclops* cf. *dengizicus* and the ciliate *Fabrea* cf. *salina*. In addition to their exceptional salinity tolerance, they were involved in a remarkably simple food web. In June 2009, a bloom of an orange-pigmented cyanobacterium (*Cyanothece* sp.) was recorded in False Bay and persisted uninterruptedly for 18 months. Stable isotope analysis suggests that this cyanobacterium was the main prey item of *F.* cf. *salina*. This ciliate was then consumed by *A.* cf. *dengizicus*, which in turn was presumably consumed by flamingos as they flocked in the area when the copepods attained swarming densities. On the shore, cyanobacteria mats contributed to a population explosion of the staphylinid beetle *Bledius pilicollis*. Although zooplankton disappeared once salinities exceeded 130, many taxa are capable of producing spores or resting cysts to bridge harsh periods. The hypersaline community was disrupted by heavy summer rains in 2011, which alleviated drought conditions and resulted in a sharp increase in zooplankton stock and diversity.

**Conclusions/Significance:**

Despite the current freshwater deprivation crisis, the False Bay region has shown to be resilient, harboring a unique biodiversity with species that are capable of enduring harsh environmental conditions. However, further freshwater deprivation may extend beyond the physiological thresholds of this community, as well as other unique biodiversity components which this system sustains.

## Introduction

Salinity is widely recognized as an important ecological factor, with potential to drastically influence the composition and dynamics of aquatic ecosystems. Salt lakes and tidal pools are often characterized by hypersaline conditions, with salinity levels exceeding about 40 and at times even reaching the saturation point of about 300 (crystallizing brine) [1]. The St. Lucia Estuary is one such estuarine lake, characterized by dramatic and intense changes in salinity, with levels ranging from near fresh-water in the southern parts of the estuary, to over 300 at times in the northern regions. It is the largest estuarine lake in Africa and forms part of the iSimangaliso (formerly Greater St. Lucia) Wetland Park, South Africa's first UNESCO World Heritage Site [2], [3]. Ecologically, it is the most important nursery ground for juveniles of marine species on the south-east African coastline, contributing to the juvenile fish population of a large area of the adjacent continental shelf [4], [5]. The area characteristically experiences cyclical wet and dry phases, each lasting between four and ten years [6]. St. Lucia is, however, currently experiencing an unprecedented crisis in terms of freshwater deprivation. This is due to below average rainfall persisting since 2002 and a range of anthropogenic interventions undertaken during the last century. Prior to 1920, the Mfolozi River discharged into the St. Lucia Estuary and would buffer water loss during periods of drought, but in the 1930s a canal was excavated through the Mfolozi flats for agricultural purposes [6], [3]. The natural filtration system of the swamps was, therefore, destroyed and the two systems have been artificially maintained separate since 1952 in an attempt to avoid the threat of siltation from the Mfolozi [3]. This alteration of the system's catchment has further exacerbated the severity of the current drought. Low freshwater input and high evaporation rates have led to the persistence of a reversed salinity gradient, with hypersaline conditions in the upper reaches, i.e. False Bay and North/South lakes. These harsh conditions combined with the closed-mouth state, have resulted in only the most adaptive species remaining [7].

Few organisms are capable of tolerating such extreme conditions, but some are so specialized that they are actually able to flourish, as competition for resources virtually disappears [8]. The branchiopod *Artemia* spp., popularly known as brine shrimps, are typical examples of this, as they are able to take over entire ecosystems worldwide, provided that salinity in the medium is high enough to exclude any other potential crustacean competitors. However, they are unable to sustain their populations at salinity levels equivalent to, or below, that of seawater, as here they are outcompeted by more generalist species [9]. Among copepods, a number of harpacticoid and cyclopoid species are known to occur in hypersaline waters around the world. For instance, *Tigrioupus californicus* has been repeatedly observed in high numbers in natural tide pools near San Francisco (USA) at salinity levels exceeding 100 [10]. Also, the cyclopoid copepod *Apocyclops dengizicus* has been collected from Australian waters with salinities up to 75 [11] and Dexter [12] was able to induce its reproduction at salinities up to 68, with adults able to survive for 120 days at 79 and 60 days at 107.

**Figure 1:**
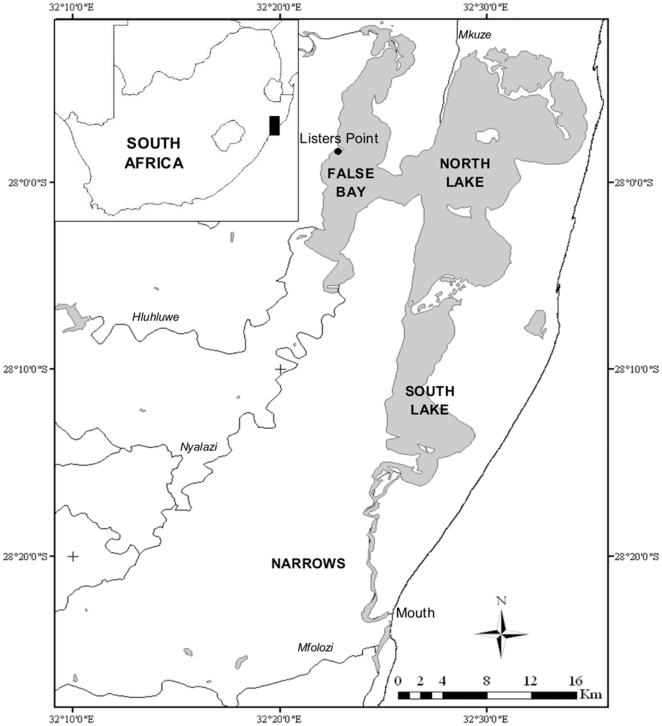
Map of the St. Lucia Estuary. The map shows the geographic location of the St. Lucia Estuary within South Africa and the sampling stations occupied during the study period.

In the St. Lucia Estuary, False Bay and North Lake are most susceptible to the current freshwater deprivation effects. The relatively mild hypersaline conditions (70–90) that developed in the North Lake between 1969 and 1971, as a direct result of a drought, led to a number of extraordinary changes in some of the basic trophic relations. These involved mainly: (1) a bloom of dinoflagellates [13]; (2) the dominance of chironomid larvae and harpacticoid copepods in bentho-pelagic samples [14]; (3) a population explosion of aerial spiders; and (4) the loss of most of the plankton present, leaving only few species with high salinity tolerance [14], [15]. The unique circumstances that the estuary is currently experiencing have provided the opportunity to investigate how communities would now respond to fluctuating environmental conditions. This study documents the changes that have occurred in the biotic communities within the False Bay region of the St. Lucia Estuary (Figure 1) under the current hypersaline crisis, with the aim of contributing a better understanding of ecosystem functioning during hypersaline conditions in extreme habitats. This information may also offer insight into how communities may respond to future environmental changes.

## Materials and Methods

### Ethics statement

Permission for this study was granted under a research agreement with the iSimangaliso Wetland Park Authority for the project titled “Climate Change and the Management of KZN Estuaries: St. Lucia Estuary”.

### Sampling frequency

Surveys were undertaken at Listers Point, False Bay, St. Lucia Estuary (Figure 1), from May 2010 through to June 2011. Sampling during 2010 occurred at irregular intervals. Samples were collected bimonthly, from 8 May to 16 July. Thereafter sampling frequency was decreased due to lake water levels being extremely low, or no water being present at all. Bimonthly samples were then resumed in January 2011, when water levels increased due to heavy rain in the catchment. In the subtropical climate of KwaZulu-Natal there are essentially two seasons, one characterized by regular rainfall (October–April) and the other with virtually no rain at all (May–September). The study period, therefore, covered both the dry season (May 2010–October 2010) and the wet season (November 2010–April 2011). Zooplankton, phytoplankton and microphytobenthic samples, together with physico-chemical data, were collected on each sampling survey.

### Physico-chemical variables

Physico-chemical measurements were taken with a YSI 6920 water quality logger, fitted with temperature, depth, conductivity, dissolved oxygen, pH and turbidity probes. In cases where the water level was less than 10 cm deep, the probe was placed horizontally so that all the sensors were submerged.

### Pelagic and benthic microalgae

Subsurface water samples were collected on each of the sampling occasions. Two 100 mL subsamples were filtered through a GF/F filter to determine the total concentration of chlorophyll *a*. Phytoplankton biomass (mg pigm.m^−3^) was determined fluorometrically (Turner Designs 10-AU) after cold extracting chlorophyll-*a* and phaeopigments from filters in 10 mL 90% acetone for 48 h in the dark. Microphytobenthic cores (2 cm internal diameter, n = 3, depth = 1 cm) were collected on each occasion and placed in 100 mL polyethylene bottles containing 30 mL 90% acetone for microphytobenthic chlorophyll *a* and phaeopigment extraction [16]. Biomass was again determined fluorometrically and expressed as mg pigm.m^−2^.

### Zooplankton

Single daytime zooplankton samples were collected using an epibenthic sled fitted with 100 µm mesh. This method was employed due to the shallow water levels and the diurnal nature of many of the zooplankton taxa present in the system [17]. The mouth of the net was semi-circular in shape (r = 18.5 cm) and mounted on a sled such that the net was raised 7.5 cm above the sediment surface. The volume of water filtered (∼1.43 m^3^) was calculated by multiplying the area of the sled mouth by the distance towed (27 m). Samples were emptied into 500 mL polyethylene bottles containing 4% phloxine-stained formaldehyde. At times when depth was too shallow, or when zooplankton was too dense for the sled to be used, 30 L of water was passed through a 100 µm sieve.

In the laboratory, samples were suspended in 1–5 L solutions, depending on the density of organisms. The main sample was then stirred vigorously so that all the organisms remained in a homogenous suspension and a 20 mL plastic vial attached to a metal rod was used to withdraw 3 subsamples from mid-depth [18], [19]. Zooplankton within the samples was identified and counted with a dissecting microscope (×400) and density was calculated as ind.m^−3^. Zooplankton taxa were identified using identification manuals [20], [21] as well as by submitting specimens for analysis to experts of the relevant groups (see Acknowledgements for list) Biomass was estimated by oven-drying (24 hours, 60°C) between 30 and 100 individuals of each taxon in pre-weighed tin capsules. Triplicate weights were obtained for each species. The average weight for each species was then multiplied by the respective abundance so as to obtain the average dry weight per sample (mg DW.m^−3^).

### Stable isotope analysis

For stable isotope analysis, sedimentary organic matter (SOM), particulate organic matter (POM), microphytobenthic biomass (MPB), macroalgae, coleopterans (*Bledius pilicollis*), fish (*Oreochromis mossambicus*) and zooplankton samples were collected from Listers Point in May 2010 and January 2011.

In order to get an estimate of sedimentary organic matter (SOM), sediment from Listers Point was collected and treated with excess 2% hydrochloric acid (HCl), in order to remove any inorganic carbonates in the form of CaCO_3_ which may have been present. Once thoroughly rinsed with distilled water, the sediment was dried in the oven at 60°C for 24 hours and subsequently crushed with a pestle and mortar. The resultant powder was packaged in microcentrifuge tubes for further processing. Thick (∼1 cm) algal mats which were present on the shoreline at Listers Point were also collected and treated in a similar manner.

For the extraction of microphytobenthos (MPB), a procedure similar to that of Couch [22] was employed (c.f. [23], [24]). Microalgae were then filtered onto previously combusted (450°C, 6 hours) Whatman GF/F filters. Water samples for particulate organic matter (POM) were also filtered onto pre-combusted GF/F filters. In May 2010, POM was divided into two fractions: <20 µm (*Cyanothece* dominant) and >20 µm (ciliate dominant) POM. All filters were then acid treated with 2% HCl. Once rinsed and dried, the filters were packaged in aluminium foil envelopes and sent to the Stable Light Isotope Unit, Department of Archaeology, University of Cape Town.

Zooplankton, which was collected with an epibenthic sled, was concentrated on 200 µm Nitex mesh and frozen prior to laboratory sorting into the dominant taxa. These were the copepods *A.* cf. *dengizicus* and *Pseudodiaptomus stuhlmanni*. Approximately 200 individuals were needed for each of the 3 replicates. In order to extract lipids, zooplankton was defatted in a solution of methanol∶ chloroform∶ distilled water (2∶1∶0.8) [25]. Once dried, the zooplankton was packaged in tin capsules for isotope analysis.

A cast net, operated from the shore was used to collect specimens of the fish *O. mossambicus*, which were frozen prior to laboratory processing. For stable isotope analysis, muscle tissue samples were excised from individual fish (in triplicate), dried at 60°C, and homogenised into fine powder using mortar and pestle.

In order to get a signal for the staphylinid beetle *B. pilicollis*, the head and abdomen of each individual were removed so as to get a pure signal, uncontaminated by gut contents. The combined thoraxes of 3 individuals were then dried at 60°C for 24 hours and subsequently packaged into 3 replicate tin capsules for isotope analysis.

Samples were weighed into 5×8 mm tin capsules and analysed by the Stable Light Isotope Unit (Department of Archaeology, University of Cape Town, South Africa). Samples were combusted in a Flash EA 1112 series elemental analyzer (Thermo Finnigan, Italy). The gases were passed to a Delta Plus XP IRMS (isotope ratio mass spectrometer) (Thermo Electron, Germany), via a Conflo III gas control unit (Thermo Finnigan, Germany). All stable isotope ratios are reported in the conventional delta (δ) notation as parts per thousand (‰) deviation from the international standard:
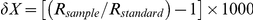
where: *X* is ^13^C or ^15^N and *R* is the corresponding ratio of ^13^C/^12^C or ^15^N/^14^N

The global standard for the carbon isotope was Vienna Pee Dee Belemnite and atmospheric nitrogen for the nitrogen isotope.

### Statistical analyses

Univariate statistical analyses were conducted with SPSS version 19 for Windows. Since data satisfied the assumptions of parametric testing (i.e. normality and even distribution of residuals), one-way analysis of variance (ANOVA) was applied to test for possible temporal differences in total zooplankton abundance, biomass and species richness. Spearman correlations were used to test for relationships between environmental variables and the three univariate community parameters.

Multivariate analysis was conducted on abundance data using the PRIMER package (Version 6.0). All data were square-root transformed. A Bray-Curtis similarity matrix was calculated from the samples collected over the study period. Cluster analysis (group averaged) was then used to visually assess spatio-temporal differences in zooplankton assemblages, and tested using analysis of similarity (ANOSIM). Where differences were found, the SIMPER routine was used to determine the relative contribution of individual species to community structure. The BIOENV function (using Harmonic Spearman Correlation) was then used to relate environmental variables to the zooplankton communities. The PRIMER software was also used to construct Abundance Biomass Comparison (ABC) curves and calculate W-statistics [26] for the surveys conducted from 10 January 2011 until 6 June 2011. The ABC method was initially proposed as a technique for monitoring disturbance on benthic invertebrate communities [27], by comparing dominance in terms of abundance and biomass. In undisturbed states, the community is supposed to be dominated by slow-growing, large, late maturing species, with the biomass curve lying above the abundance curve. With increasing disturbance, the slow-growing species are outcompeted by fast-growing, small, opportunistic species, resulting in the biomass curve falling underneath the abundance curve. The W-statistic is the difference between the two curves, with a negative sign indicating a disturbed community [28].

## Results

### Physico-chemical variables and microalgal biomass

**Figure 2:**
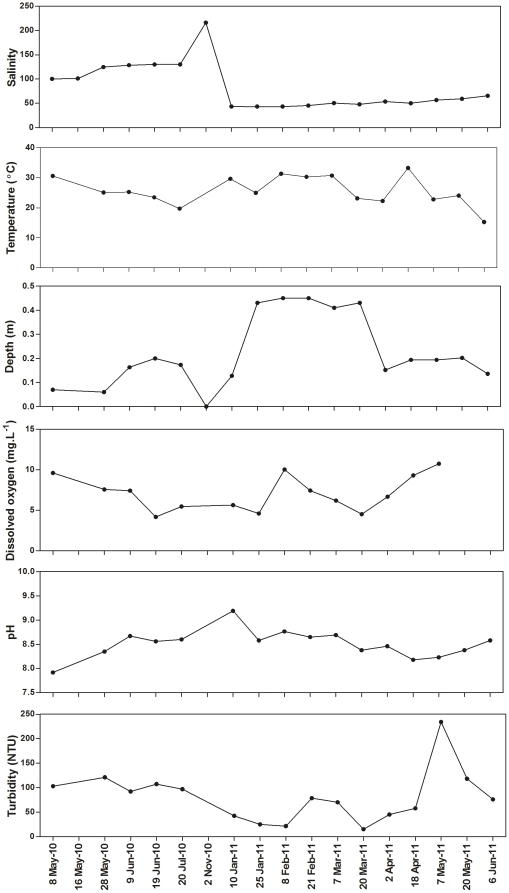
Physico-chemical variables measured at Listers Point during the study period. Limited measurements were taken in November 2010 due to lack of water, while logistic reasons prevented the collection of all physico-chemical data on 16 May 2010.

The study period covered essentially 2 different phases, as reflected in the physico-chemical conditions measured. A hypersaline phase persisted from May to November 2010. Up until July, salinity levels exceeded 100 and water levels were generally less than 0.2 m in depth. From July to November 2010, however, the False Bay region was almost completely dry and when there was water available, salinity levels exceeded 200. The second phase included a period of high rainfall. The heavy summer rains of December 2010 and January 2011 diluted the hypersaline waters down to 43.5 and increased water levels substantially, to an average of 0.5 m. At the end of the rainy season, however, salinity levels increased incrementally over time, reaching 66 by the end of the study period, in June 2011, while water levels dropped to about 0.25 m in average depth (Figure 2).

Water temperatures ranged from 15.3°C, on 6 June 2011, to 33.3°C, on 18 April 2011. Dissolved oxygen was highly variable throughout the study, with values ranging from 4.18 mg.L^−1^ on 19 June 2010 to 10.8 mg.L^−1^ on 7 May 2011. Turbidity levels were also highly variable, ranging from 15.1 NTU (Nephelometric Turbidity Units) in March 2011 to 234 NTU in May 2011 (Figure 2).

**Figure 3:**
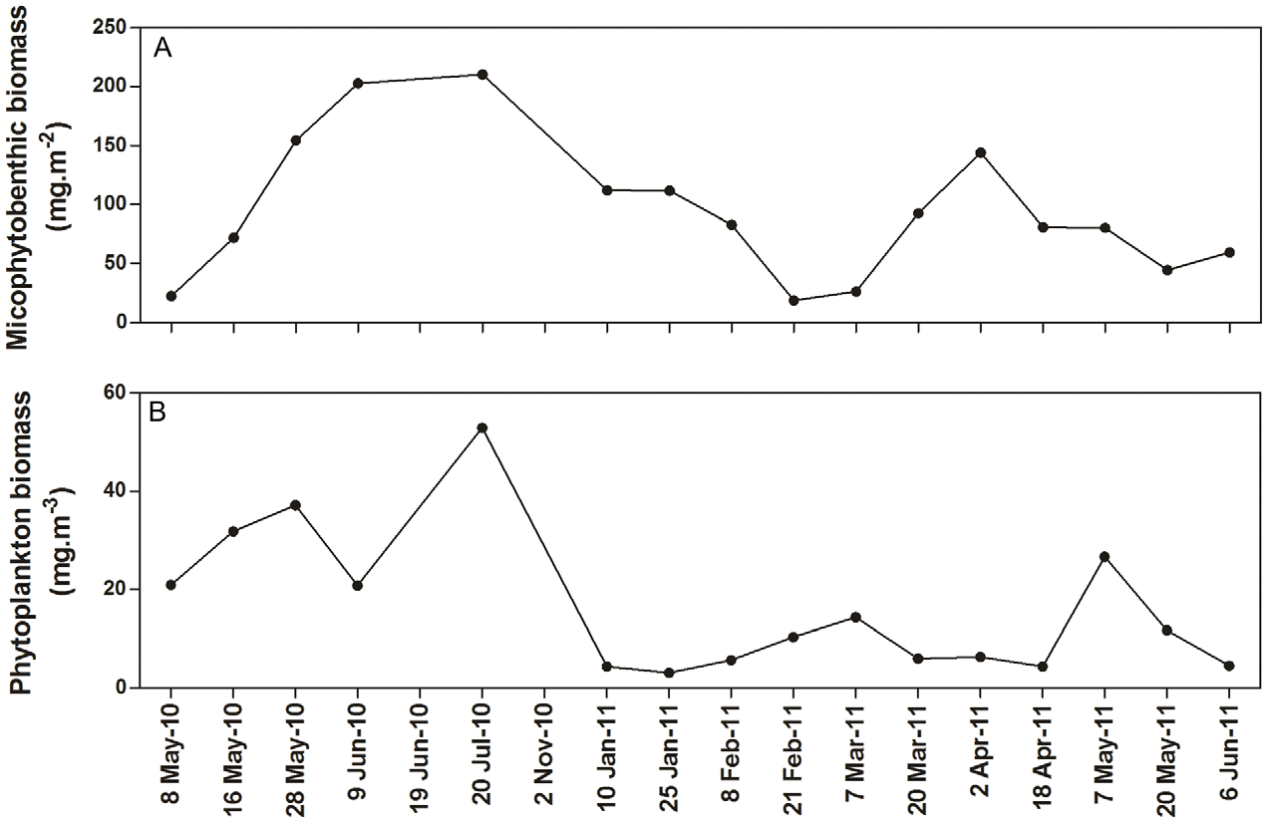
Time series of microalgal biomass at Listers Point. Microphytobenthic (A) and phytoplankton (B) biomass levels measured at Listers Point throughout the study period.

Regarding microalgae, phytoplankton biomass ranged from 3.12 mg pigm.m^−3^ in January 2011 to 52.9 mg pigm.m^−3^ in July 2010, while microphytobenthic biomass ranged from 18.9 mg pigm.m^−2^ in February 2011 to 210 mg pigm.m^−2^ in July 2010 (Figure 3).

### Zooplankton community structure, abundance and biomass

**Figure 4:**
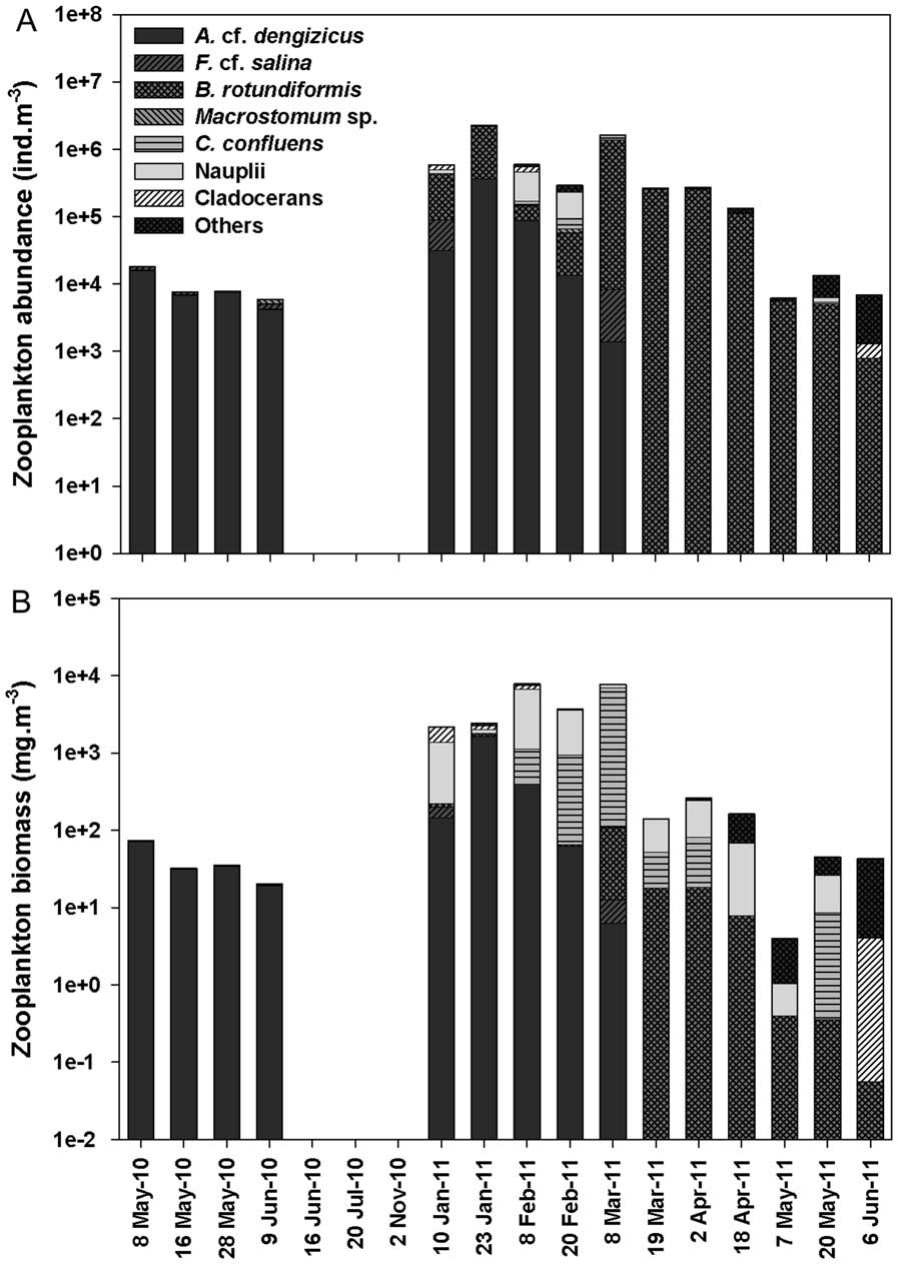
Time series of zooplankton abundance and biomass at Listers Point. Average zooplankton (A) abundance and (B) biomass of the dominant taxa recorded at Listers Point during the study period.

Total recorded zooplankton abundance ranged from 5.9×10^3^ ind.m^−3^ in June 2010 to 2.3×10^6^ ind.m^−3^ in January 2011 (Figure 4A). The highest zooplankton densities corresponded with the increased freshwater inflow received in early 2011. Similarly, zooplankton biomass peaked with increased freshwater input, reaching a maximum of 7.9 g dw.m^−3^ (Figure 4B). Both zooplankton abundance and biomass were significantly higher in the wet season than in the dry season (ANOVA, Abundance: F*_1,16_* = 6.71, *p*<0.05; Biomass: F*_1,16_* = 5.77, *p*<0.05). There was, however, no significant difference in species richness between seasons (ANOVA, F*_1,16_* = 3.94, *p*>0.05). Spearman's rank correlation between environmental parameters and zooplankton density and biomass identified significant correlations. In particular, zooplankton abundance and biomass correlated negatively with salinity, turbidity, temperature and phytoplankton biomass (Table 1). For zooplankton species richness on the other hand, a significant negative correlation was only found with salinity and phytoplankton (Table 1).

**Table 1:** Spearman's rank correlation between environmental parameters and zooplankton abundance, biomass and species richness at Listers Point.

		Temp.	Salinity	DO	pH	Depth	Turbidity	PPL	MPB
Abundance	Coefficient	**0.52**	**−0.89**	−0.004	0.34	0.43	**−0.71**	**−0.64**	−0.27
	*p*	0.04*	0.00***	0.99	0.2	0.09	0.00**	0.01*	0.30
	N	16	16	14	16	16	16	15	15
Biomass	Coefficient	0.57	**−0.88**	0.10	0.41	0.48	**−0.72**	**−0.61**	−0.37
	*p*	0.03*	0.00***	0.73	0.11	0.06	0.00**	0.02*	0.18
	N	16	16	14	16	16	16	15	15
Species richness	Coefficient	0.41	**−0.69**	0.26	0.24	0.49	−0.47	**−0.58**	−0.51
	*p*	0.12	0.00**	0.37	0.38	0.06	0.07	0.02*	0.05
	N	16	16	14	16	16	16	15	15

Values in bold indicate significant correlations. MPB: microphytobenthic biomass, PPL: phytoplankton, Temp: temperature, DO: dissolved oxygen,

*: p<0.05,

**: p<0.01,

***: p<0.001.

**Figure 5:**
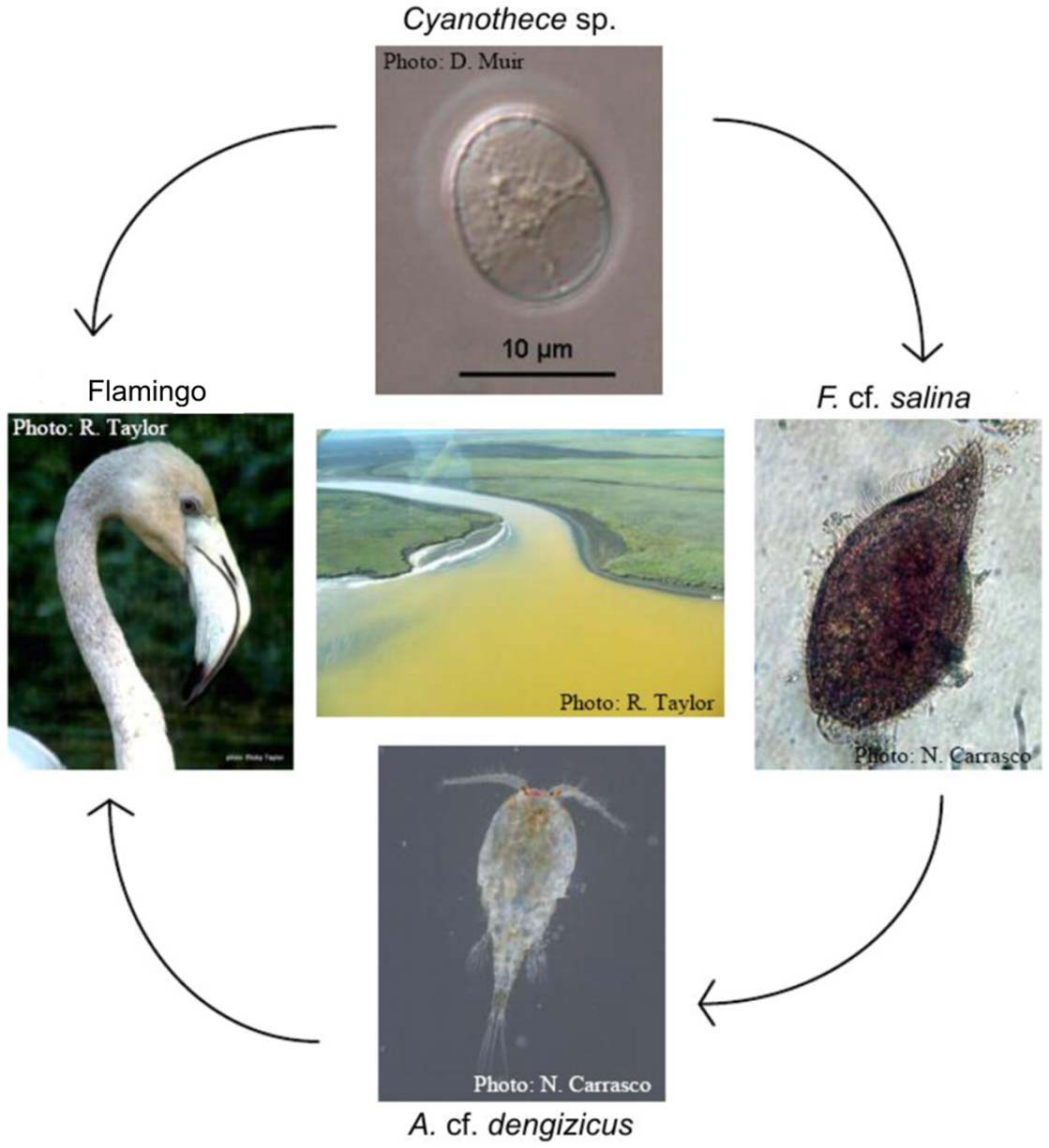
Simple food web at False Bay. Diagram representation of the simple food web observed at False Bay from May to June 2010.

**Figure 6:**
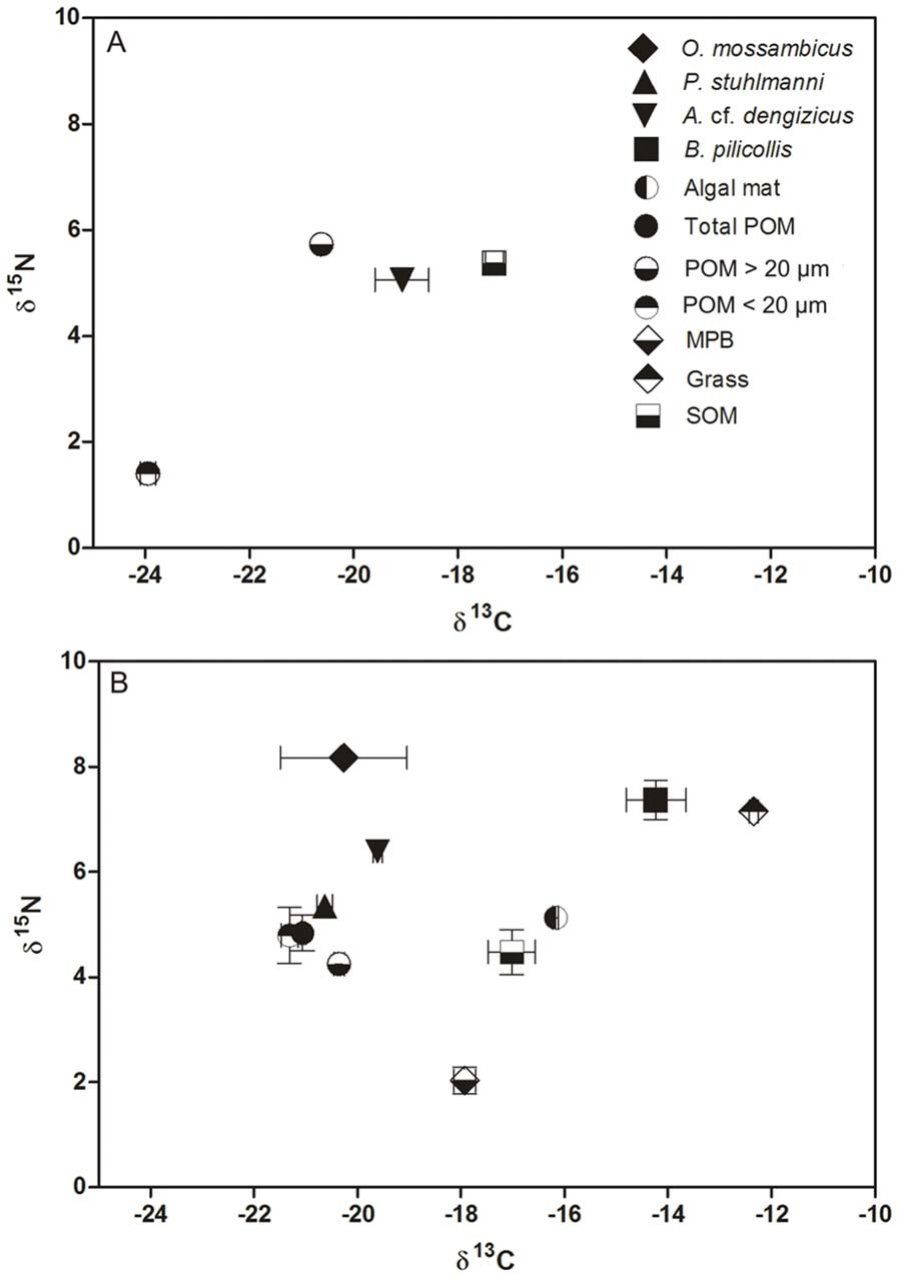
Isotopic signatures of consumers and their preys at Listers Point. Plot of δ^15^N and δ^13^C signatures of primary carbon sources and their consumers measured from Listers Point in (A) May 2010 and (B) February 2011.

A total of 15 different zooplankton taxa were recorded during the study period in False Bay. Only 4 taxa were able to withstand the harshest environmental conditions, when salinity rose above 100. These were the flatworm *Macrostomum* sp., the ciliate *Fabrea* cf. *salina*, the harpacticoid copepod *Cletocamptus confluens* and the cyclopoid copepod *A.* cf. *dengizicus*. While *C. confluens* and *Macrostomum* sp. also showed remarkable salinity tolerance (>100), they were not always present throughout the period (May–June 2010) and were found in much lower densities than *F.* cf. *salina* and *A.* cf. *dengizicus*. These two dominant species were involved in a remarkably simple “one species-per-level food chain” or alternatively a “simple food web” (Figure 5). In June 2009, a bloom of an orange-pigmented cyanobacterium (*Cyanothece* sp.) was recorded in False Bay, which persisted uninterruptedly for at least 18 months afterwards. Stable isotope signatures for POM<20 µm (*Cyanothece*-dominated) [29] and POM>20 µm (*Fabrea*-dominated) showed average carbon values of 23.9 and 20.6‰, respectively, indicating that the cyanobacterium was the main prey item for *F.* cf. *salina* (Figure 6A). This ciliate was then consumed by *A.* cf. *dengizicus*, which eventually led to Greater and Lesser flamingos flocking to the area when the copepods attained high densities (R. Taylor, pers. comm.) (Figure 5). Once salinity levels exceeded 130, no zooplankton was recorded in the region. The heavy summer rains that fell in December 2010/January 2011, however, reduced drastically salinity levels and increased water depth, resulting in the reappearance of the aforementioned species, as well as high densities of the rotifer *Brachionus rotundiformis* and the cladocerans *Diaphanosoma excisum* and *Moina micrura* (Figure 4A).

On the lake shores, *B. pilicollis* beetles emerged in huge numbers on the western shores of False Bay in January 2011. A white sheet of 4×3 m positioned at night under a 250 W mercury-vapour lamp was rapidly covered with hundreds of thousands of *B. pilicollis* individuals on 30 January. This staphylinid beetle has a δ^13^C signature of −14.2‰, which is similar to the signatures of both algal mats (−16.2‰) and grasses (−12.4‰) that are found on the shoreline. In the water-column, the Mozambican Tilapia *O. mossambicus* had the highest δ^15^N signature of 8.71‰ and its carbon values were within the range of those recorded for particulate organic matter (POM) and zooplankton (Figure 6B).

**Figure 7:**
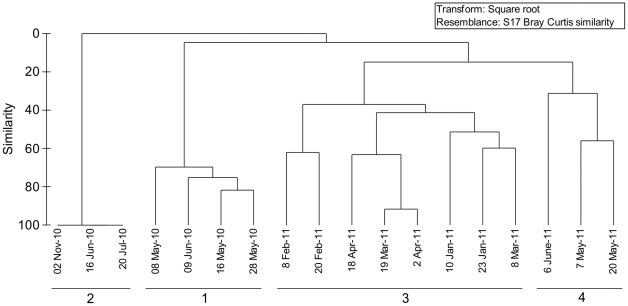
Zooplankton communities at Listers Point. Dendrogram showing the grouping of zooplankton communities observed at Listers Point during the study period.

Cluster analysis grouped zooplankton communities into 4 groups (Figure 7), which were confirmed to be significantly different by ANOSIM (R = 0.99, p<0.01). The dominant taxa in group 1 were *A.* cf. *dengizicus* and *F.* cf. *salina*, together contributing >90% of the total zooplankton abundance. No zooplankton was recorded from the period 16 June to 2 November 2010, explaining the 100% similarity shown in group 2. Group 3 was characterized by taxa, such as *B. rotundiformis*, *C. Confluens*, *A.* cf. *dengizicus* and copepod nauplii, which collectively contributed over 90% of the total zooplankton abundance. The last grouping included the samples collected between 7 May and 6 June 2011. During this period, salinity levels exceeded 60 and the zooplankton community was dominated by species such as *A. natalensis*, *P. stuhlmanni*, *B. rotundiformis* and polychaete larvae.

The BIOENV procedure identified different environmental variables influencing the plankton communities. Overall, the interactions between salinity and phytoplankton biomass best explained the patterns observed in the zooplankton assemblages (R = 0.798). On its own though, salinity was the single most important variable influencing zooplankton communities (R = 0.692).

**Figure 8:**
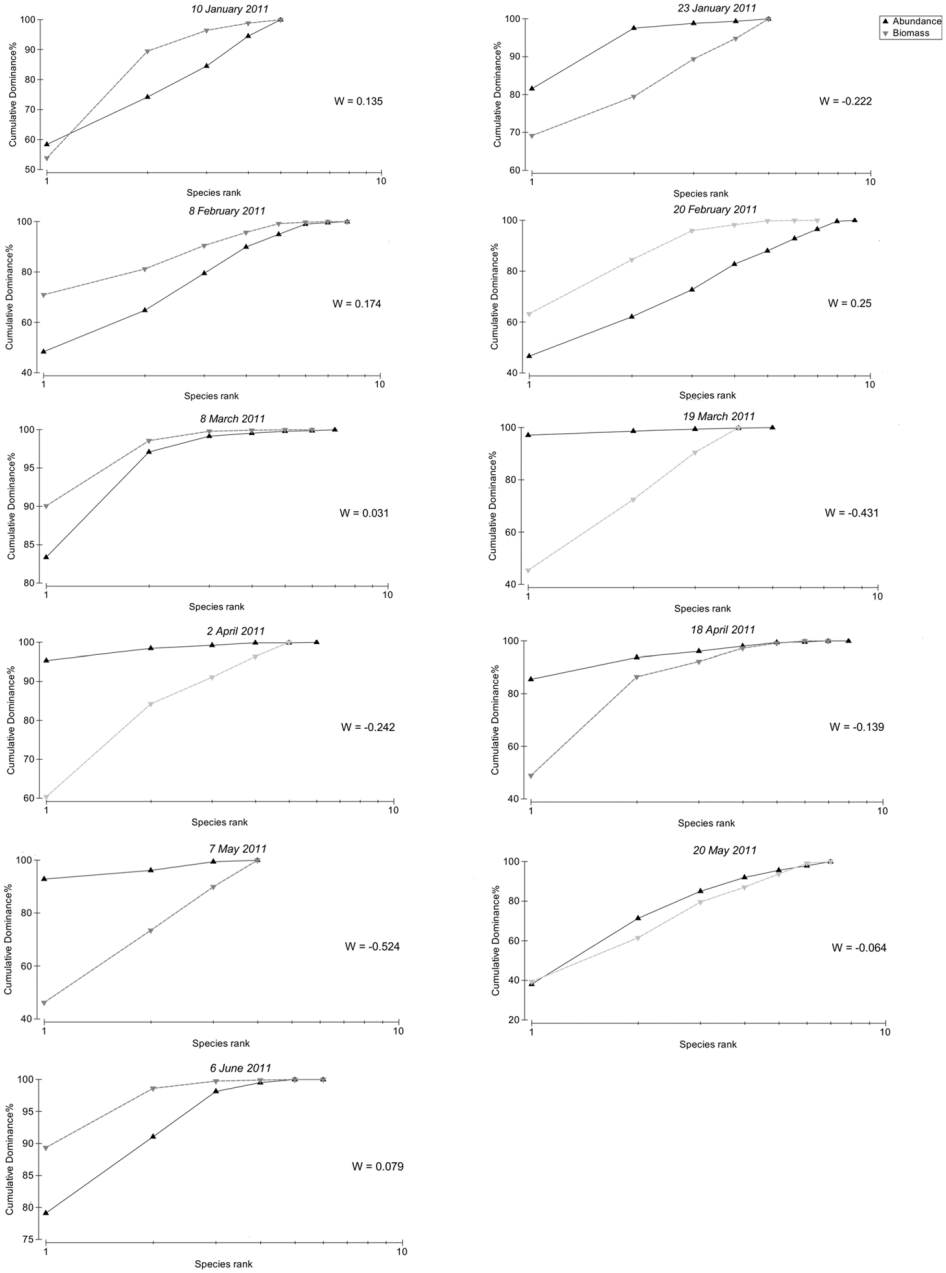
Abundance-Biomass Comparison (ABC) curves. Figure shows the ABC curves generated for surveys conducted from 10 January to 6 June 2011 at Listers Point.

**Figure 9:**
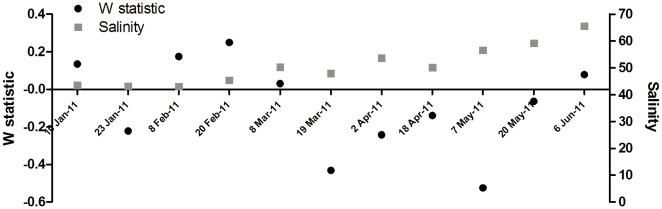
Time series of W-statistics at Listers Point. Trend in the W-statistic for the period January to June 2011 at Listers Point.

Figure 8 shows the ABC curves for the surveys conducted from 10 January to 6 June 2011. At the beginning of 2011, with the exception of 23 January 2011, the biomass curves lie above the abundance curves and have positive W-statistics. Thereafter, however, the W-statistics become more negative with the biomass curves falling below the abundance curves for each survey until 20 May 2011. At this time the two curves cross and the W-statistic becomes once again positive by 6 June 2011. Figure 9 provides the temporal trends in the W-statistic for each of the surveys plotted against the salinity levels. Although the levels differ slightly between surveys, the general temporal trend shows a decline in habitat health with time in conjunction with the increase in salinity.

## Discussion

Extreme events are increasing worldwide, in response to climate change [30], [31] and the unprecedented hypersaline event recorded at St. Lucia during this study is largely an expression of this phenomenon (e.g. [3]). The nature of freshwater deprivation in the St. Lucia Estuary has resulted in a northward gradient of drought effects. While regions in the south have recently been relatively protected from the drought, due to freshwater input from the Mpate and Mfolozi Rivers through the link canal [3], hypersalinity and low water levels have become increasingly more severe towards the north. Shallow lake levels, coupled with low freshwater input, may at times lead to salinity levels in excess of 200 [32]. However, despite the dynamic nature of the environment and the rapidly changing physico-chemical parameters, the False Bay region has shown substantial resilience until recently.

In this study both zooplankton abundance and biomass were significantly higher during the wet season, compared to the dry hypersaline phase. This is consistent with findings reported in the literature and may be linked to the increased phytoplankton production brought about by the increased nutrient load associated with freshwater input [33]. In this study, phytoplankton biomass correlated negatively with zooplankton abundance. This is most likely a product of high grazing rates, with high zooplankton densities resulting in larger grazing impact, thereby reducing phytoplankton biomass. A negative correlation also existed between zooplankton abundance/biomass and salinity, as well as turbidity, reflecting the negative impact of both hypersaline and turbid conditions on zooplankton stock. Species richness was also negatively affected by increases in salinity, a relationship which is common in hypersaline systems [34]. The high summer rainfall received in December 2010 and January 2011 diluted the estuarine water from a salinity >200 to <50, creating a habitat that was more conducive to zooplankton life, since the upper salinity tolerance limit of most estuarine zooplankton species at St. Lucia is around 60 [35]. Increased freshwater input, therefore, not only increased zooplankton abundance and biomass, but species richness as well. The BIOENV procedure identified different environmental variables influencing the zooplankton communities. Overall, the interactions between salinity and phytoplankton biomass best explained the patterns observed in the zooplankton assemblages. On its own though, salinity came out as the single most important variable influencing zooplankton communities.

The False Bay region supported only a small zooplankton community during 2010, as only 4 taxa were able to withstand the harsh environmental conditions. These were the flatworm *Macrostomum* sp., the harpacticoid copepod *C. confluens*, the cyclopoid copepod *A.* cf. *dengizicus* and the ciliate *F.* cf. *salina*, with both potentially new and undescribed species (F. Fiers and W. Petz, pers. comm.). All four taxa have demonstrated remarkable salinity tolerance, only disappearing from the region once salinity levels exceeded 130. Records of the same or related species in hypersaline conditions have been reported elsewhere in the literature. The heterotrichous ciliate *F. salina* is physiologically well adapted to high salinities [36] and is, therefore, a typical pelagic dominant in hypersaline environments [37], [38]. Elloumi et al. [39] recorded *F. salina* at salinities ranging from 80 to 300. It was suggested though, that its distribution within extreme environments is closely related to the abundance of its prey [39]. *F. salina* also has a competitive advantage over other species, as it is capable of producing a mucilaginous substance that inhibits the growth of other halotolerant species, including the different life-cycle stages of the anostracan *Artemia*, a known competitor of *F. salina* ([40] and references therein). *A. dengizicus* has been collected from Australian waters with salinities up to 75 [11]. Dexter [12] was able to induce reproduction at salinities up to 68 and adults were able to survive for 120 days at 79 and 60 days at 107. The tolerance limits recorded for *A.* cf. *dengizicus* during this study exceeded those previously observed. It is either possible that the slow acclimation occurring under natural conditions increased their tolerance, or this species has some other method of withstanding these extreme hypersaline waters. It is also possible that this may actually be a new, undescribed species/subspecies with higher tolerance limits than those observed in *A. denzigicus* (F. Fiers, pers. comm.). Individuals collected in the field at 130 were highly inactive, only responding to mechanical stimulus. Subsequent decreases in salinity led to increased individual activity (N.K. Carrasco, pers. obs.). A dormancy period is common within zooplankton [41], allowing many species e.g. cladocerans, copepods, rotifers and ciliates to bridge seasonally unfavourable conditions. Dormancy in zooplankton can be subdivided into quiescence and diapause. Quiescence is a phase of retarded development, allowing a fast response to changing environmental conditions, while diapause is a period of ‘deep sleep’, which once initiated is only terminated by certain intrinsic and/or environmental stimuli [42]–[45]. It is likely that *A.* cf. *denzigicus* at St. Lucia entered a phase of quiescence during the unfavourable hypersaline conditions, which would have increased its tolerance range.

In addition to having some of the highest recorded salinity tolerances for invertebrates, during the study period, *A.* cf. *dengizicus* and *F.* cf. *salina* were involved in a remarkably simple food web. In June 2009, a bloom of the orange-pigmented cyanobacterium *Cyanothece* sp. was recorded in False Bay, persisting uninterruptedly for at least 18 months [29]. This bloom is believed to have been initiated by high levels of nutrients made available by the decomposition of organisms, which died because they were unable to withstand the harsh conditions that prevailed in the region during the previous months. Stable isotope evidence suggests that this cyanobacterium was the main prey item of *F.* cf. *salina* from May to July 2010. This ciliate in turn became prey to the cyclopoid copepod *A.* cf. *dengizicus*. Cyclopoid copepods are primarily raptorial [46] and are known to be omnivorous [47], preferentially feeding on soft-bodied defenseless organisms such as slow moving ciliates [48]–[52]. Tiffany et al. [53] also demonstrated the ability of *A. dimorphus* to control large ciliate populations through the strong inverse relationship found between *A. dimorphus* densities and that of large ciliates. Predation by *A.* cf. *dengizicus* would also explain the inverse relationship between itself and *F.* cf. *salina* densities documented in this study. Although stable isotope signatures were not obtained for flamingos, anecdotal evidence suggests that *A.* cf. *dengizicus* was consumed by Greater and Lesser flamingos which flocked in the area when the copepods attained swarming densities (R. Taylor, pers. comm.). While Greater flamingos feed mostly on zooplankton, Lesser flamingos feed almost entirely on cyanobacteria, the pigment from which accumulates in their feathers, giving them the typical pink colouration [54]. As is often the case with isotopic studies, it is not always possible to get a signal for all available carbon sources. It is possible that other organisms, such as *Macrostomum* sp. and *C. confluens* for instance, (which although present, were not in high enough abundance to provide an isotopic signature) were at the time contributing marginally to this food web. The term “one-species-per-level food chain” or “simple food web” is, therefore, used tentatively, with the knowledge that the organisms specified above (*Cyanothece* sp., *F.* cf. *salina*, *A.* cf. *dengizicus* and flamingos) were the overwhelming components of the food web, and not necessarily the only ones involved in it.

Once salinity levels exceeded 130, no zooplankton was recorded in the region. It was hypothesized though, that these species are capable of producing spores or resting cysts, capable of surviving unusually harsh conditions for long periods of time. It has been shown that during hypersaline conditions, sediments may harbor a potentially rich biodiversity in the form of dormant species, which is not apparent by sampling the water-column alone [8]. The heavy summer rains of December 2010/January 2011 helped alleviate some of the drought conditions, resulting in the reappearance of the aforementioned species, as well as high abundances of the rotifer *B. rotundiformis* and the cladocerans *D. excisum* and *M. micrura* as well as the harpacticoid copepod *Nitocra taylori* sp. nov., a new endemic species currently under description [55].

Also associated with the *Cyanothece* bloom was the occurrence of thick (up to ∼1 cm) algal mats, largely made up by cyanobacteria (several taxa) and diatoms (D. Muir, pers. comm.). While the mat formation was probably not linked to the *Cyanothece* bloom per se, the reasons for their extensive occurrence (high nutrient loads as well as low grazing pressure) might be the same. Under windy conditions, these algal mats were lifted off the estuarine bed and deposited on the shoreline where they likely performed an important ecological trophic role. In January 2011, once salinity levels decreased, huge densities of *B. pilicollis* beetles were recorded at False Bay. Adults were attracted in their thousands by a mercury-vapour lamp deployed on the shores of the lake and in less than two hours a 4×3 m sheet was covered entirely by beetles. It was suspected that their larvae may be growing on this decaying algae. Species of *Bledius* are commonly found in unvegetated or lightly vegetated, sunny, moist sand adjacent to rivers, lakes, and oceans. Adults and larvae are known to feed on algae and diatoms that live in the moisture surrounding each sand grain. Their habitat is selected based on soil moisture, salinity, texture, and, indirectly at least, size of the sand grains, availability of food, and amount of shade [56]. Stable isotope results in this study suggested that *B. pilicollis* may indeed be feeding on the algal mats as their carbon signatures were very close together. The signature for grass, which was collected from the adjacent shoreline was, however, also very close to that of *B. pilicollis*. It is, therefore, most likely that this beetle was feeding on a combination of algal mats and terrestrial vegetation.

The zooplankton communities of the Salton Sea bear remarkable similarities with those currently found in the northern Lakes of the St. Lucia Estuary. The Salton Sea is an enclosed saline lake situated in the Colarado Desert of southern California and exhibits average salinity levels around 44 [57]. Although these levels are significantly lower than those found in the upper reaches of the St. Lucia Estuary, similar taxa are found in both systems. The Salton Sea fish community, like that of St. Lucia, has also become overwhelmingly dominated by the euryhaline Mozambican Tilapia, *O. mossambicus* [58]. Under hypersaline conditions, *O. mossambicus* is able to out-compete both estuarine residents and estuary-associated marine fish spawners [59]. In St. Lucia, however, there have been a number of fish kills which have been attributed to cold temperatures (R. Taylor pers. comm.), since this species, having an optimal temperature range of 20–35°C, is unable to tolerate temperatures below 13°C [60]. Caskey et al. [58] also documented periodic die offs of this species, attributing them to the combined effects of fluctuations in temperatures and dissolved oxygen levels, high salinity, toxic algal blooms and parasite infestation. Given the opportunistic feeding mode that this key species exhibits, it is obviously capable of having significant effects on ecosystem size and structure. An experiment with Salton Sea micro-ecosystems showed that at realistic densities, the tilapia caused large reductions in amphipod and corixid populations, increases in certain harpacticoid, rotifer, nematode and ciliate populations as well as an increase in periphyton and decrease in phytoplankton abundances [61], [62]. It is, therefore, likely that *O. mossambicus* also played a role in structuring zooplankton communities at False Bay, since *O. mossambicus* was the main predator in the region and communities here were dominated by taxa similar to those recorded in the Salton Sea study.

The St. Lucia Estuary has experienced a number of hypersaline events in the past, although none of those appeared to have been as severe as the current one [3]. The hypersaline conditions (70–90) that developed in its North Lake between 1969 and 1971, as a direct result of a previous drought, led to a number of extraordinary changes in some of the basic trophic relations [13]. The high salinities and low water levels associated with freshwater deprivation resulted in mass mortality of fauna. In terms of the plankton, only those species with relatively high salinity tolerance, such as *P. stuhlmanni* and *Acartiella natalensis* remained [15]. Once the salinity levels dropped down to 34–42, the large amount of organic nutrients which was then readily available, coupled with still calm conditions, resulted in the proliferation of the dinoflagellate *Noctiluca scintillans* in the northern lakes. This was the first record of a red tide in a South African estuary and was unique in that it occurred 50–60 km from the estuary mouth [13]. An associated phenomenon was the dominance of chironomid larvae (*Chironomus kaffrarius*) and harpacticoid copepods in bentho-pelagic samples [14]. These swarms were also favoured by the calm conditions, but were most likely exacerbated by the mortality of their normal predators in the lakes during the preceding drought. In addition to this, myriads of aerial spiders belonging to the family Tinyphiidae were found feeding on the chironomids. It was reported that in the False Bay region, spider webs became so dense that plants and branches of trees were completely smothered and killed [13]. These events to some extent resemble those which were witnessed at False Bay during this study, in that a cyanobacterial bloom resulted in unique changes in the trophic functioning of the system. Since the environmental conditions experienced in the early 1970s were very similar to those described in this study, the system was expected to respond in a similar manner. However, this was not the case. Not only were the organisms involved in this study completely different from those documented by Grindley and Heydorn [13], but most of them had also not been previously recorded in the St. Lucia Estuary [35]. This is an indication that the system has indeed changed significantly over time.

The False Bay region has shown to be quite resilient, harboring a unique biodiversity with species that are capable of enduring harsh environmental conditions both through euryhaline adaptations as well as the ability to form resting stages or spores. However, the results of the ABC curves, and their corresponding W-statistic for the zooplankton communities, suggest that the local assemblages do become increasingly stressed with increased freshwater deprivation. This shift towards a more disturbed state can be inferred from the temporal trend in the W-statistic, which was assessed from January 2011, when high rainfall was received, through to June 2011. Although the levels differ slightly between surveys, the general temporal trend shows a decline in habitat health with time in concurrence with the increase in salinity. However, by 6 June 2011, the W-statistic was once again positive suggesting that above a salinity threshold, a resident hypersaline community may develop, involving species that are capable of thriving under these conditions. However, while the False Bay region has shown to be fairly resilient, further freshwater deprivation may extend beyond the physiological thresholds of even this community, as well as other unique biodiversity components which this system sustains
